# Body image drawings dissociate ethnic differences and anorexia in adolescent girls

**DOI:** 10.1186/s13034-017-0150-y

**Published:** 2017-03-14

**Authors:** Galit Goldzak-Kunik, Micah Leshem

**Affiliations:** 1 Nutrition Unit, Department of Diabetes, Haifa and Western Galilee, Clalit Health Services, Lin Medical Center, Rothschild Street 37, Haifa, Israel; 2 Department of Psychology, University of Haifa, 31905 Haifa, Israel

**Keywords:** Adolescent girls, Anorexia nervosa, Body image dissatisfaction, Body image figure drawings, Ethnic differences

## Abstract

**Objectives:**

To distinguish between ethnic differences among segregated schoolgirls and restrictive anorexia nervosa using a simple culture-fair test of body image (BI) figure drawings.

**Methods:**

Several responses to BI figure drawings by 178 adolescent schoolgirls from three ethnically distinct and segregated schools and communities in Israel, Jewish secular (JS), Jewish Haredi (H), and Christian Arab (C), and a group of 14 severely restricting anorexic girls (AN). BI evaluations were analyzed by MANCOVA, followed by paired or Student-t tests for comparisons between responses and groups respectively. Pearson r served for correlations and the Fisher Z for differences between slopes.

**Results:**

Despite the total ethnic segregation among the schoolgirls, there are commonalities; all prefer a thinner ideal BI, and are similarly dissatisfied with their BI. However, ethnic differences also emerge: C underestimate their BI and how others view them, and H true and Ideal BI evaluations correlate, unlike the other groups. Despite this variability, and in stark contrast, the anorexic girls show a gross misperception of their BI, even in comparison to girls equated for BMI.

**Discussion:**

The findings show that figure drawings evaluation of BI is a simple and robust instrument dissociating clinical and ethnic responses. Clinicians may consider body figure drawings as a simple, supportive, diagnostic for first-line recognition for risk of AN in adolescent girls.

## Background

Eating disorders (ED) are among the most common psychiatric disorders in young women. “A registered dietician may be the first to recognize an individual’s ED symptoms or be the first health care professional consulted by a patient for this condition” [1] and “early identification is crucial because shorter duration of illness is associated with improved outcome in ED” [2]. “the presentation of eating disorders is often cryptic—for example, via physical symptoms in primary care. The ability to diagnose the condition varies and can be inadequate, and existing questionnaires for detection are lengthy and may require specialist interpretation” [3]. The 5-question SCOFF is intended for non-specialist use but its utility for the general population and specifically for adolescents is uncertain [4, 5]. Hence, simple diagnostic aids are continually sought.

In multi-ethnic communities it is often helpful to distinguish between cultural norms and potential diagnostic features, particularly because cultural factors influence body weight norms, weight control, obesity, and body image (BI), body image dissatisfaction, and consequently may both obscure or increase vulnerability for ED [6–18]. At the same time in anorexia nervosa (AN), BI dissatisfaction is an essential diagnostic and together with the drive for thinness is extreme and underlies and perpetuates the psychopathology [8, 15, 19, 20].

Women, especially adolescent, are more likely to experience BI concerns [2, 7, 9, 18, 21, 22]. Social norms of feminine beauty, particularly western norms of shape and body weight are consistently correlated with increased weight consciousness and risk of ED [14–16, 21, 23, 24]. However, most studies of ethnic differences in risk factors for ED have examined largely intermingled ethnicities, often in the same institution, exposed to similar media, and with little segregation. In addition, questionnaires used across cultures, often in translation, may not be understood as intended [12].

In Israel, distinct ethnic communities have been studied in relation to propensity for ED. Indigenous Moslem, Druze, Circassian, Christian and Bedouin schoolgirls, as a group in the north of Israel, score higher on ED questionnaires than their Jewish peers, while within the group Circassians score lower and Bedouin higher. Among the Jews, Kibbutz girls scored higher in one study, but not another, and religious girls lower. Such differences are often attributed to socio-cultural traits, ‘modernity’. and exposure to western media [6–8, 13–16, 19, 24].

What makes Israel multi-ethnicity different, and somewhat unique, is the complete segregation of different cultural groups often in adjacent habitation. Ethnic and religious identities may be profoundly segregated by residential area, education (schools and colleges separated by ethnic and religious makeup and teaching programs), language, degree of traditionality and religiosity, exposure to different mass media or none at all, basic values and attitudes toward femininity, sex roles, marriage and divorce, family relations, child-rearing, dietary restrictions, and more [7, 12–17]. Such profound demarcations may place an even greater onus on ED tests to reliably distinguish between the cultural and clinical. Thus, in 10 distinct groups of Israeli schoolgirls, EAT-26 subscales in at least 3 of the groups overlapped with AN scores [15].

Hence it is of interest to enquire whether segregated ethnicities differ in BI perception, whether such differences can be evaluated without recourse to language and translation, and whether such differences might overlap and compromise clinical evaluation of BI distortion [8]. Thus, tests that can screen for risk of ED, are robust across ethnicities and culture-fair, are of interest, especially if simple and brief [5, 8, 13].

Here we examined the robustness of a simple instrument (body image figure drawings [23]) to distinguish between cultural and clinical differences in evaluating BI. We compared three groups of ethnically diverse and segregated schoolgirls, and a group of severely restricting adolescent AN girls. Christian Arab girls (C) and Jewish secular girls (JS) differ in cultural, traditional, and religious content and norms, access distinct media differing in language (Arabic and Hebrew), with the Arabs tending to the more traditional. Jewish Haredi girls (H) also differ in language, using both Yiddish and Hebrew, are deeply religious and traditional, and are totally isolated from media (radio, TV, theatre, film, internet, non-sectarian magazines and newspapers, and books (other than scriptures)], and State educational programs [10, 12, 13].^1^

The three groups are entirely separated in and out of school and have negligible or no knowledge of each other.

Among Arab girls, Christians score lower on ED symptoms, but score higher than Jewish girls [7, 12, 17]. Among Jewish girls, the Orthodox score lower, and their self-esteem relates positively to religious fervor and negatively to ED scores, the authors suggesting that religiosity provides protection from ED [13, 25]. Haredis are considerably more religious, traditional, and segregated than the Orthodox Jewish stream, possibly predicting even lower ED scores for the H girls [9, 10].

Such differences are often attributed to socio-cultural traits and exposure to western media [7, 11–18, 24], although there have been few empirical studies relating specific cultural traits and ED [18, 24].

Our aim was to examine whether a simple test would distinguish the marked ethnic differences between these three groups and to what extent they might overlap those of AN.

## Methods

### Participants

Ethics approval was obtained from the Ministry of Education and the School Head for the schoolgirls. Girls and parents signed informed consent forms sent from the school. No incentives were offered. The researchers explained the selection criteria to the School Head: grade, sex (girls—Haredi schools are not mixed), no ED prior or current, and no weight change greater than 5 kg in the previous 6 months. The Head scheduled the classes for the study, and in effect, all the girls in the selected classes volunteered. Testing was carried out by two research assistants in Hebrew or Arabic. The schoolgirls completed the questionnaire with the figure drawings at their desks in the classroom. Subsequently, in a separate room, individually, they were weighed and height measured. To reduce the possibility of ED, schoolgirls reporting weight changes greater than 5 kg in the previous 6 months, or who were currently counselled by a clinical dietitian, were excluded (6 girls).

For AN, ethics approval was obtained from their hospital and the university. Parents and daughters gave informed consent and permission for access to relevant medical information which included BMI, because we did not weigh or measure height to avoid stress. In addition, to circumvent the possible conflictual nature of the AN-therapist relation, we emphasized to the participants that our researchers were not involved in their treatment, and were from an unrelated university (in Haifa, a different town). To minimize variability in diagnosis, treatment regimen, and environment, we enlisted participants from the same closed ward, in their first episode, and similarly diagnosed with restrictive anorexia with no comorbidities. Girls were tested within 2.9 ± 0.1 day of hospitalization to minimize entrenchment of AN behavior patterns often acquired from veteran AN patients, and prior to any recovery. Drug therapy commenced at intake and included olanzapine, amisulpride, or risperidone. Fourteen girls meeting the criteria were enrolled, 3 refused. They completed the BI questionnaire with the figure drawings individually in a separate room [20]. No incentive was offered but after the tests an unanticipated token gift of a necklace (value ~$4) was offered in appreciation.

Participant group size, age and BMI are presented in Table 1.

**Table 1:** Participant data (±SD)

	Age (years)	BMI	n
Restrictive anorexic (AN)	16.0 ± 1.1*	17.3 ± 1.8*	14
Jewish Secular (JS)	14.2 ± 1.3	19.4 ± 3.9	81
Christian Arab (C)	14.9 ± 0.4	22.1 ± 2.9*	37
Haredi (H)	14.5 ± 1.3	20.1 ± 3.3	60

Different from other groups, * p’s < 0.05–0.001

### Body image evaluation

Using 7 different schematic figure drawings of girls [23], participants marked a continuous linear scale below the images in response to each of 3 questions: (1) the image “most like you”, (2) their “ideal body shape”, and (3) “which figure would others consider most like you” (in Hebrew, or verbally in Arabic).

### Statistical analysis

For analysis, scores were converted to age-corrected BMI in the 3rd–97th percentiles (BMI%) (26). BI Dissatisfaction was calculated as the difference between questions 1 and 2. BI evaluations were analyzed by MANCOVA with BMI% and age as covariates, followed by paired or Student-t test for comparisons between responses and groups respectively (SPSS 19). Dissatisfaction was separately evaluated by univariate analysis because it is not independent of the questions.

Means are presented with standard deviation, and effect size is the partial eta squared (PES). Pearson r served for correlations and the Fisher Z for differences between slopes.

## Results

MANCOVA for the 4 groups and 3 questions with BMI% and age covariates shows a group effect, F (9558)=4.3, p < 0.001, PES = 0.065, due to question 1, “most like you”, F (3186) = 10.8, p < 0.001, PES = 0.149, and 3, “others consider most like you”, F (3186) = 8.8, p < 0.001, PES = 0.124. The girls do not differ in their thinner Ideal (Table 2). Univariate analysis with BMI% and age covariates for dissatisfaction shows a group effect, F (3187) = 2.9, p < 0.05, PES = 0.045, due to AN more dissatisfied than schoolgirls, whereas it does not differ among schoolgirls. However, 1-sample tests show that all are dissatisfied with their BI (p’s < 0.001. Table 2).

**Table 2:** BI self-evaluation expressed as BMI% compared to true BMI%

	True BMI%	Question 1. Most like you	Question 2. Ideal shape	Question 3. Others consider most like you	Dissatisfaction (questions 1–2)
AN	15.3 ± 15.6	58.8 ± 17.1***	30.4 ± 12.0*	55.4 ± 20.0***	28.4 ± 28.5
JS	52.1 ± 26.2	50.5 ± 15.3	41.9 ± 11.7***	47.1 ± 20.9	8.7 ± 17.1
C	66.7 ± 26.9	50.6 ± 17.0***	39.5 ± 12.6***	48.4 ± 18.0***	11.1 ± 16.5
H	48.4 ± 29.8	51.0 ± 17.0	39.1 ± 12.2*	48.3 ± 20.0	11.8 ± 16.2
Thin	10.13 ± 6.3	34.1 ± 15.3^++^	38.0 ± 13.6^+^	31.4 ± 15.9^++^	−3.9 ± 14.7^+++^

Responses to a series of schematic body image drawings thin to obese

*AN* anorexics, *JS* Jewish secular, *C* Christian Arab, *H* Haredi, schoolgirls

Differ from true BMI%: * p < 0.05, *** p < 0.001

Thin schoolgirls matched to AN for BMI%. AN differ from Thin: ^+^ p < 0.05,^++^ p < 0.005,^+++^ p < 0.001. Thin girl evaluations also diverge significantly from true BMI% (not indicated)

**Fig. 1:**
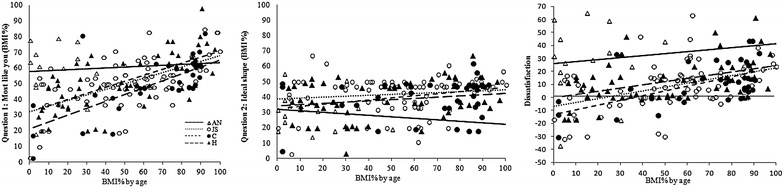
BMI% estimates by the girls for each of the 3 questions against ‘true’ BMI%

For a detailed analysis of trends between the groups we compared slopes of BI scores. Self-estimation of body size correlates with BMI% in groups JS, C and H but not in AN. Ideal preference correlates with BMI% only in H, and dissatisfaction correlates with BMI% in schoolgirl groups (p’s < 0.001) but not in AN, who are more dissatisfied (Fig. 1; Table 2).

Left panel: body size estimates correlate for the schoolgirls (JS, 0.61, C, 0.70, H, 0.67, p’s < 0.001) but not for AN. AN differ from the other slopes (Fisher Z, JS = −2.06, C-2.33, H = −2.33, p’s < 0.05). Center panel: ideal estimates correlate significantly only for H (0.33, p = 0.01). Slopes do not differ significantly. Right panel, dissatisfaction with own BI (difference between questions 1 and 2). Dissatisfaction correlates for the schoolgirls, (JS, 0.45, C, 0.58, H, 0.46, p’s < 0.001), but not for AN (0.08). The slopes do not differ. Horizontal reference line denotes ‘no Dissatisfaction’ and below it—‘Satisfaction’.

Finally, to isolate any effect of low BMI we directly compared AN responses with 29 schoolgirls selected for BMI% <25 (‘Thin’). MANCOVA for the 2 groups and 3 questions with BMI% and age as covariates shows a group effect F (3,37) = 4.6, p < 0.01, PES = 0.273, due to group effects on question 1, “most like you”, F (1,39) = 9.1, p < 0.005, PES = 0.189, question 2, “Ideal shape”, F (1,39) = 4.3, p < 0.05, PES = 0.099, and questions 3, “others consider most like you”, F (1,39) = 10.7, p < 0.005, PES = 0.215 (Table 2). Univariate ANOVA confirmed that Dissatisfaction differs between AN and Thin, F (1,39) = 13.5, p < 0.001, PES = 0.257, because Thin girls are not dissatisfied with their BI (Table 2). Like the other schoolgirls, Thin girl evaluations diverge significantly from true BMI% (data not shown).

## Discussion

In comparing 3 groups of schoolgirls we find that despite substantive ethnic differences, BI evaluations are similar: Ideal shape is thinner than true BMI%, and dissatisfaction significant.

Ethnic differences in BI evaluations also emerged. Although C slightly underestimate their BI, show a greater disparity between true BMI%, and their thinner Ideal, and believe that others see them as thinner than they are, this might be an artefact of their greater BMI. Traditional Arab society viewed plumpness as desirable [12, 15], but we doubt that these girls were still influenced because their Ideal is similar to that of their peers, and they are equally dissatisfied, suggesting that their greater disparity between true BMI%, and their thinner Ideal reflects an adaptive BI. Our finding that C do not differ in body dissatisfaction from JS and other ethnic groups is supported by assessments using the EDI-2 [7, 12], although there were higher scores among the Arab girls in other ED-2 subscales that were suggested to be due to Arab women’s stressors and difficulties in a patriarchal community rather than specific eating or thinness ideals [17].

The correlation of BMI% and Ideal preference in H is not found in the other groups. Although like the other girls their ideal was thinner, and dissatisfaction similar, the correlation suggests that the thinner ideal was determined by their own BI, i.e. each girl would like to be thinner in relation to her own BI rather than to an externally determined ideal (as by media exposure, to which H are not exposed). Conversely, because the ideal for the other schoolgirls is unrelated to their BI, it may derive from an external, consensual ideal, consistent with the belief that media exposure contributes to the desire for thinness by its incessant portrayal as beauty [6, 7, 9, 11, 12, 18, 21, 22, 24].

Haredi women conform to strict dress codes, almost uniform clothing concealing arms and legs in public, and wigs to conceal hair or their shaved head. The religious rationale is to minimize attraction to men, and may therefore also diminish the importance of BI [10]. Along with media isolation, religion, and culture, this may account for less ED in this and other traditional communities [7, 10, 13, 16, 25].

In stark contrast, AN show a gross misperception of their BI. Unlike the healthy schoolgirls, there is no relationship between dissatisfaction and BMI percentile among AN (r = 0.08, Fig. 1 right panel) [19–21] who are thus dissatisfied with their BI irrespective of its true dimensions. We now show that, again, unlike the schoolgirls, this misperception extends to believing that others also see them as much bigger than they are. As repeatedly reported and we confirm here, they overestimate their size, are clearly dissatisfied with their BI, and their Ideal is thinner [8, 19, 20, 22].

Finally, a direct comparison with equally thin, but healthy, schoolgirls confirms the clear difference in BI evaluation. A thin healthy girl has positive body satisfaction, and her ideal is only slightly heavier than how she sees herself. This is important because it suggests that body figure drawings might be a simple instrument to help first-line clinicians recognize adolescent girl patients presenting with, eg, weight loss and high BI dissatisfaction, at risk for ED.

### Limitations of the study

The AN group was small because of the difficulty of finding AN homogenous by age, diagnosis (severely restricted) and recency of hospitalization, as our AN participants were. Ethnically they were Jewish secular, however, in all measures they clearly differed from the healthy JS, C and H schoolgirls including those matched for BMI. AN were also older but the differences survived statistical adjustment for age.

## Conclusions

We show that BI evaluation with figure drawings, while sensitive to ethnic differences, is robust across adolescent girls of segregated ethnicities and media exposure. On the other hand, the test clearly distinguishes between ethnic variation and restrictive anorexia nervosa.

Clinicians may consider body figure drawings for simple first-line recognition and referral for risk of AN in adolescent girls.

Although these preliminary results are suggestive, validation by extensive prospective studies would be of interest.

## Abbreviations

AN: anorexia nervosa
BI: body image
C: Christian
ED: eating disorder
JS: Jewish secular
H: Haredi
BMI%: BMI percentile by age
PES: partial eta squared

## Footnotes

1 Jews (including Haredis) are ~80% of the population, Christian Arabs ~0.016%, and Haredis ~9.9%. Statistical abstract of Israel 2010. Demographic characteristics. Population, by population group, religion, sex and age http://www.cbs.gov.il/reader/shnaton/templ_shnaton_e.html?num_tab=st02_19&CYear=2010. Accessed 02 April 2016. Wikipedia, the free encyclopedia. Demographics of Israel. https://en.wikipedia.org/wiki/Demographics_of_Israel#Ethnic_and_religious_groups. Accessed 02 April 2016.
